# Scaffolding proteins in G-protein signaling

**DOI:** 10.1186/1750-2187-2-13

**Published:** 2007-10-30

**Authors:** Alexandra V Andreeva, Mikhail A Kutuzov, Tatyana A Voyno-Yasenetskaya

**Affiliations:** 1 Department of Pharmacology, College of Medicine, University of Illinois at Chicago, 909 S. Wolcott Ave, Chicago, Illinois 60612, USA

## Abstract

Heterotrimeric G proteins are ubiquitous signaling partners of seven transmembrane-domain G-protein-coupled receptors (GPCRs), the largest (and most important pharmacologically) receptor family in mammals. A number of scaffolding proteins have been identified that regulate various facets of GPCR signaling. In this review, we summarize current knowledge concerning those scaffolding proteins that are known to directly bind heterotrimeric G proteins, and discuss the composition of the protein complexes they assemble and their effects on signal transduction. Emerging evidence about possible ways of regulation of activity of these scaffolding proteins is also discussed.

## Background

The concept of scaffolding (or scaffold) proteins, formulated more than a decade ago (see [1] for an early review), is now widely accepted in different areas of cellular signaling. Scaffolding proteins simultaneously associate with two or more partners, thus providing specificity of signaling and enhancing the interactions between components of a particular signaling module [2,3]. It should be mentioned that scaffolding proteins may not necessarily only function as "passive" anchoring platforms for "active" components of the signaling complex. Some scaffolding proteins may directly affect the activity of proteins they bind.

Heterotrimeric G proteins (recent reviews: [4,5]) are ubiquitous signaling partners of seven transmembrane-domain G-protein-coupled receptors (GPCRs), the largest (and most important pharmacologically) receptor family in mammals. In addition to their role in signal transduction from canonical GPCRs, heterotrimeric G proteins are also involved in signaling downstream of a number of "non-canonical" receptors [6] and signaling events that do not employ transmembrane receptors at all [7]. While the number of G protein-coupled receptors in mammalian cells exceeds 1000, the number of heterotrimeric G proteins is much smaller, even taking into account the number of possible combinations of α, β and γ subunits. In the recent years, it has become clear that scaffolding proteins may provide not only signaling efficiency, but also specificity to G protein-coupled signaling.

This review aims to address the variety of scaffolding proteins that interact with heterotrimeric G proteins and the diversity of the signaling complexes they assemble. Proteins that belong to the classes of regulators (RGS) and activators (AGS) of heterotrimeric G-protein signaling, many of which can also act as scaffolds, have been recently covered in other reviews, and the reader is referred to the respective recent publications (RGS proteins: [8,9]; AGS proteins: [10,11]).

## AKAPs

A-Kinase Anchoring Proteins (AKAPs) are a family of scaffolding proteins that target PKA and other signaling enzymes to specified subcellular locations [12,13]. Each AKAP contains a conserved amphipathic helix responsible for high affinity binding to the dimer of the regulatory (R) subunits of PKA [14,15], and a targeting domain that directs the PKA-AKAP complex to specific subcellular compartments [16-20]. Due to their ability to interact with several signaling proteins, AKAPs bring PKA in a close proximity with a variety of signaling partners [21-25]. Several AKAPs play a role in various aspects of G protein-coupled receptor signaling [26], and at least two of them directly interact with heterotrimeric G proteins.

**Figure 1:**
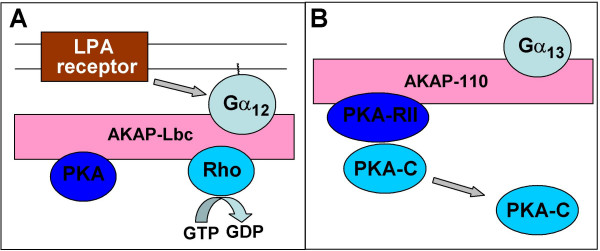
Schematic representation of Gα_12_/Gα_13_-containing signaling complexes assembled by A-kinase anchoring proteins (AKAPs): AKAP-Lbc (A) and AKAP-110 (B). See main text for details and references.

*AKAP-Lbc *was identified as a PKA-anchoring protein highly expressed in the heart that also has a Rho-specific guanine nucleotide exchange (GEF) activity ([27,28] reviewed by [29]). It was shown to interact with Rho in the inactive GDP-bound or nucleotide free form, but not with activated GTP-bound Rho. AKAP-Lbc was found to mediate activation of Rho by mutationally activated Gα_12 _and, to a lesser extent, by Gα_13_, but not several other tested Gα [28]. Moreover, AKAP-Lbc was shown to interact with Gα_12 _and to mediate Gα_12_-dependent Rho activation by lysophosphatidic acid (LPA) receptor ([28]; Fig. 1A) and by α_1 _adrenergic receptor, in the latter case mediating cardiomyocyte hypertrophy [30]. Anchored PKA phosphorylates AKAP-Lbc, thus allowing binding of 14-3-3, which in turn inhibits its RhoGEF activity by interfering with the interaction between AKAP-Lbc and RhoA [31,32]. Inhibition of RhoGEF activity by 14-3-3 requires AKAP-Lbc oligomerization [33]. These findings imply that cellular cAMP levels would regulate sensitivity of Gα_12_-dependent signaling *via *AKAP-Lbc. AKAP-Lbc may also anchor PKCα, which might phosphorylate Rho or Rho GDP dissociation inhibitor (RhoGDI) although its precise role in the AKAP-Lbc complex is unknown [24].

AKAP-Lbc also assembles an activation complex for the lipid-dependent enzyme protein kinase D (PKD) [24]. It contributes to PKD activation in two ways: it recruits the upstream kinase PKCη and coordinates PKA phosphorylation events that release activated protein kinase D. Thus, AKAP-Lbc synchronizes PKA and PKC activities in a manner that leads to the activation of a third kinase, PKD [24].

Notably, in both cases (Gα_12_-Rho and PKA/PKC-PKD signaling) AKAP-Lbc receives signals from G protein-coupled receptors. It is not yet clear whether the same AKAP-Lbc complex may involve simultaneously Gα_12_/Rho and PKC/PKD and process these signals in parallel, or AKAP-Lbc/Gα_12_/Rho and AKAP-Lbc/PKC/PKD represent separate and independent signaling complexes.

*AKAP110*, expressed mainly in testis, was found to selectively interact with activated Gα_13_, but not Gα_12 _or other Gα [34]. Unlike AKAP-Lbc, AKAP110 does not affect GDP-GTP exchange by Rho (J. Niu and T. Voyno-Yasenetskaya, unpublished data). It also has no effect on GDP-GTP exchange and GTPase activity of Gα_13 _[34]. Gα_13 _binding to AKAP110 does not affect binding of the regulatory subunit of PKA, but leads to a release of the catalytic subunit (Fig. 1B). Thus, AKAP-110 appears to be a Gα_13 _effector that provides a cAMP-independent mechanism of PKA activation.

Interestingly, the PKA-binding site of AKAP110 is also able to bind ropporin [35]. Ropporin is known to bind rhophilin, which is a Rho binding protein and presumable Rho effector [36]. Thus, although activation of PKA by Gα_13 _*via *AKAP110 is Rho-independent, it cannot be excluded that AKAP110 may provide yet undefined additional link between Gα_13 _and Rho signaling.

*AKAP120*. Yet another PKA-anchoring protein, AKAP120, has been suggested to specifically interact with Gα_13 _but not several other Gα [37]. However the initial finding using the yeast two hybrid system has not been confirmed by other techniques, and no further studies of the Gα_13_-AKAP120 interaction have been reported. AKAP120 described in Ref. [37] is actually a fragment of one of the several alternatively spliced isoforms derived from the same gene, which include AKAPs 350, 450 and Yotiao. These proteins are located at centrosomes and/or Golgi apparatus and affect microtubule dynamics (AKAP350/450), or regulate cardiac potassium channel (Yotiao) [38,39]. It would be important to establish whether any of these isoforms are able to interact with Gα_13_.

## Tetratricopeptide repeat (TPR)-containing scaffolding proteins

**Figure 2:**
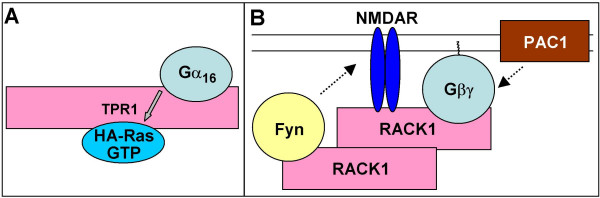
Signaling complexes assembled by TPR-repeat scaffolding proteins: TPR1 (A) and RACK1 (B). See main text for description.

*TPR1 *was initially identified as a protein containing three TPR motifs, which interacts with the GAP domain of neurofibromin (NF1) [40]. It also interacts with and is a co-chaperone of Hsp70 [41,42]. TPR1 was found to interact with Gα_16_, as well as Gα_q_, Gα_s_, and to a much lesser extent with Gα_i _(but not Gα_13 _or Gα_t_), as well as with HA-Ras (but not Rho, Cdc42 and Rac) [43]. In the absence of Gα_16_, HA-Ras was found to bind to TPR1 preferentially in the activated conformation, however in the presence of Gα_16 _binding was similar for activated and inactive HA-Ras. Binding of Gα_16 _facilitated binding of HA-Ras to TPR1 and led to accumulation of the activated Ras (Fig. 2A), apparently by stabilizing the activated conformation, rather than by promoting GDP-GTP exchange. In contrast, binding of HA-Ras had no effect on the binding of Gα_16_. The physiological role of the formation of the Gα_16_-TPR1-Ras complex remains to be explored.

Mammalian AGS3 and LGN proteins and their *Drosophila *homologue Pins, which contain N-terminal TPR domains and C-terminal domains with GoLoco motifs, act as scaffolds for Gα_i _and microtubule binding protein NuMA [44,45]. This signaling complex plays a role in regulation of cell polarity and has been considered in detail elsewhere [7,10,11].

Another TPR protein, protein Ser/Thr phosphatase PP5, is able to interact with Gα_12 _and Gα_13 _[46]. It remains to be seen whether the ternary complexes exist that include PP5, Gα_12/13 _and other proteins, or binding of Gα_12/13 _and other proteins to the TPR domain of PP5 is mutually exclusive, i.e. whether PP5 can be considered as a *bona fide *scaffolding protein.

## WD40 repeat proteins

Gβ belongs to the family of WD40 repeat proteins [47]. Gβγ acts as a scaffold itself by facilitating interaction between activated GPCRs and Gα, and also by recruiting GRK2 (β-ARK) to those GPCR that are phosphorylated by this kinase [48]. It also initiates signal transduction by recruiting other scaffolding or adaptor proteins. In yeast, as discussed below, it recruits a scaffolding protein Ste5p from the cytosol to the cell membrane, where it assembles a signaling complex containing the components of the MAPK cascade.

In mammalian cells, Gβ has been found to interact with other WD40 repeat proteins, namely RACK1 (Receptor for Activated C Kinase 1), dynein intermediate chain and an uncharacterized protein AAH20044 [49-51]. Of these three proteins, only Gβγ-RACK1 interaction has been characterized in detail to date. RACK1 shares 57% amino acid similarity with Gβ1 Unlike Gβ, RACK1 does not have an N-terminal extension for binding Gγ. Although RACK1 does bind Gβγ, it does not interact with the Gα βγ heterotrimer (except that of transducin) or with Gα.

Binding of RACK1 to Gβγ affects some but not all Gβγ-mediated functions: while activation of phospholipase C β2 and adenylyl cyclase II are inhibited, Gβγ-mediated chemotaxis and activation of MAP kinase cascade are not affected. RACK1 does not affect signal transduction through the Gα subunits of Gi, Gs or Gq.

The association with Gβγ induces translocation of RACK1 from the cytosol to the plasma membrane. This has been suggested to be a mechanism to recruit PKC and other RACK1-binding proteins to the membrane [49,50]. In particular, Gβγ-RACK1 mediated PKC recruitment to GPCRs could promote their efficient deactivation by this kinase, in a manner similar to the recruitment of GRK2 by Gβγ (see above). In addition, a number of Gβγ effectors, such as Ca^2+ ^channels, phospholipase C β2 and adenylyl cyclase, as well as several Gα and RGS proteins can be regulated by PKC. Whether regulation of these proteins is subject to fine tuning by the Gβγ-RACK1 interaction has yet to be explored experimentally.

Another possibility is that the Gβγ-RACK1 complex might build larger signaling complexes, employing scaffolding properties of both Gβγ and RACK1. In this respect, it is of particular interest that some proteins bind to/are regulated by both Gβγ and RACK1. These include dynamin-1, involved in GPCR internalization; Src, a tyrosine kinase involved in Gβγ-mediated MAPK activation, and β-integrin ([52] and references therein).

Importantly, RACK1 is able to homodimerize [53], a property that may further expand its ability to form signaling complexes as schematically depicted in Fig. 2B. A model suggested by the latter study implicates a RACK1-based signaling complex in regulation of NMDA receptor (which is an ion channel) by a GPCR PAC1. According to the model of [53], in the inactive state one molecule of RACK1 in the dimer would bind NMDA receptor and link it to PAC1 *via *Gα βγ trimer, while the other PACK1 molecule would bind to a kinase Fyn, keeping it in a proximity to NMDA receptor but nevertheless preventing it from phosphorylating NMDAR (Fig. 2B). Upon PAC1 activation, the signaling complex is postulated to dissociate and released Fyn to phosphorylate and thus activate NMDA receptor [53]. It should be noted however that this model requires the ability of RACK1 to associate with G protein heterotrimer, which in most cases is not supported by experimental evidence (see above).

*Kelch repeat proteins*, although unrelated in their primary structure to WD40 repeats, form similar β-propeller structures that also mediate protein-protein interactions [54]. Two "Gβ-mimic" proteins, Gpb1 and Gpb2, containing kelch repeats rather than WD40 repeats, have been found to interact with a Gα subunit Gpa2 in yeast [55,56]. Notably, there are no known canonical Gβγ interacting with Gpa2. Unlike canonical Gβγ, Gpb1 and Gpb2 do not facilitate, but on the contrary inhibit coupling of Gpa2 with its cognate GPCR. Whether interaction of Gα with Kelch repeat proteins is unique for yeast or similar interactions may exist in mammals remains to be explored.

## Other scaffolding proteins

*Radixin *is a member of the ezrin-radixin-moesin (ERM) protein family that anchor actin filaments to the cell cortex and membrane proteins, and function in Rho signaling. ERM proteins are involved in such cytoskeleton-dependent processes as cell morphogenesis, migration and growth (reviewed [57,58]). Not surprisingly, ERM proteins may be involved in tumor metastasis [59,60]. ERM proteins are negatively regulated by an intramolecular interaction between the N- and C-terminal domains, and their ability to bind actin and other proteins is stimulated when this interaction is disrupted and the binding sites on radixin are unmasked [57].

**Figure 3:**
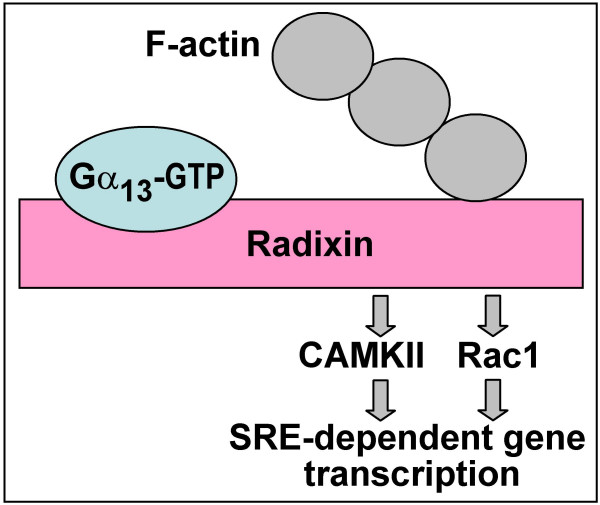
Signaling from the Gα_13_-radixin complex.

Radixin was found to interact with activated (but not GDP-bound) Gα_13 _*via *its N-terminal domain [61]. This interaction apparently disrupted the interaction between the N- and C-terminal domains, leading to radixin "conformational activation" and increased binding to F-actin [61]. Dominant-negative deletion mutants of radixin could block transformation induced by mutationally activated Gα_13_, suggesting that radixin could act downstream of Gα_13 _in this process [61]. A suggestion that radixin may mediate cellular effects of Gα_13 _was further substantiated by a more recent study from our laboratory, which found that the C-terminal domain of radixin activates Rac1 and CAMKII, leading to stimulation of SRE (serum response element)-dependent gene transcription ([62]; Fig. 3).

**Figure 4:**
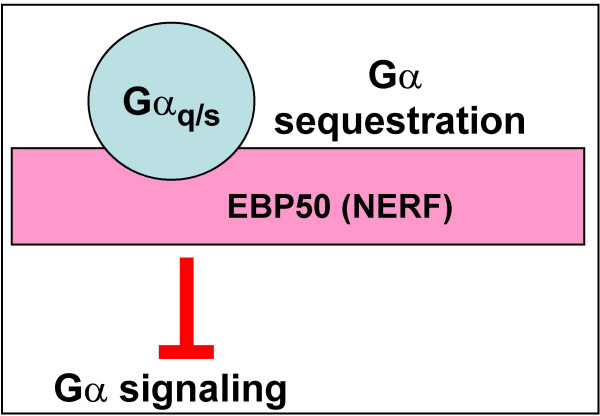
The role of EBP50 (NERF) in downregulation of G protein signaling.

*EBP50 *(ezrin-radixin-moesin-binding phosphoprotein 50, also termed NHERF1 for Na^+^-H^+ ^exchanger regulatory factor 1) is a PDZ domain-containing scaffolding protein known to bind to a variety of proteins, including ERM proteins, channels, receptors, cytoskeletal elements, and cytoplasmic proteins (for a recent review, see [63]). EBP50 was found to interact with Gα_q _*via *its PDZ domains [64]. The interaction was more efficient with activated than with inactive form of Gα_q_; accordingly, stimulation of the thromboxane A_2_receptor (which is coupled to G_q_) enhanced the Gα_q_-EBP50 interaction. EBP50 was also able to interact with Gα_s_, and stimulation of the β_2_-adrenergic receptor (coupled to Gα_s_) was found to promote EBP50 binding to Gα_s_. However, several other Gα tested (Gα_i/*o*/*t*/*z*_, Gα_12/13_, and Gα_16_) did not interact with EBP50. Sequestering of Gα_q _by EBP50 was found to interfere with the Gα-receptor interaction and to inhibit Gα_q_-dependent stimulation of PLCβ ([64]; Fig. 4). EBP50 is known to bind directly and to mediate recycling of a number of receptors [65,66]; it was also found to inhibit internalization of the thromboxane A_2_β receptor induced by Gα_q _[67]. One of the future challenges would be to establish whether, in addition to blocking Gα interactions with receptors and effectors, Gα-EBP50 interaction may functionally link Gα_q _and/or Gα_s _to other interacting partners of EBP50.

EBP50 has been found to directly interact with RACK1, another scaffolding protein capable of binding Gβγ (see above) [68]. This interaction was found to be involved in PKC-dependent regulation of an epithelial chloride channel cystic fibrosis transmembrane regulator (CFTR) [68]. Whether the ability of EBP50 and RACK1 to interact with each other is relevant to their ability to bind subunits of heterotrimeric G proteins is not known.

*Scaffolding subunit of the protein phosphatase 2A (A subunit, PR65) *was found to interact with Gα_12 _(but not related Gα_13_), which stimulated the activity of the catalytic PP2A subunit bound to the complex independently of the activation state of Gα_12 _[69,70]. Intriguingly, the same scaffolding protein PR65 was found to interact with protein phosphatase 5 (PP5) [71], and PP5 was shown to interact with and to be activated by Gα_12 _and Gα_13 _[46]. It would be interesting to establish whether PR65 may play a role in assembling the Gα_12/13_-PP5 complex as well.

**Figure 5:**
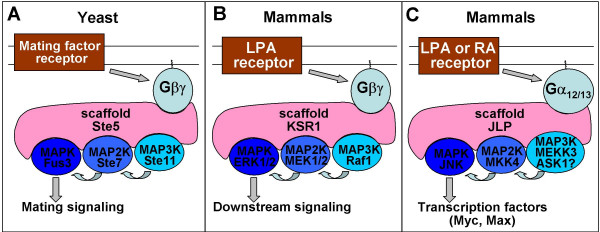
Scaffolding proteins that link subunits of heterotrimeric G proteins with MAP kinase cascade. A, The role of Ste5 in the mating signaling in yeast. B, Signaling complex assembled by KSR1, leading to ERK activation. C, Signaling complex assembled by JLP, leading to activation of JNK (or p38, see discussion in the main text). LPA, lysophosphatidic acid; RA, retinoic acid.

*Scaffolding proteins that link heterotrimeric G proteins with MAP kinase cascades*. The first such scaffolding protein, Ste5, was discovered in yeast (reviewed by [72,73]). Ste5 is able to bind all three kinases (MAPKKK, MAPKK and MAPK) of the mating pathway, which promotes their mutual interactions and thereby facilitates signal transmission. Ste5 also binds to Gβγ. The latter property allows the recruitment of the Ste5/MAPK signaling complex to the stimulus-sensing receptor/G protein module, enabling signal transduction from GPCR *via *Gβγ to the MAPK cascade (Fig. 5A). Analogs of Ste5 act as scaffolds for other MAP kinase cascades in both yeast and mammalian cells [74,75], and some of them are also able to interact with subunits of heterotrimeric G proteins.

Mammalian KSR1 protein (which shares no sequence similarity with Ste5) assembles the kinases of the ERK pathway (Raf-1, MEK1/2, ERK1/2) (reviewed by [74,76]). KSR1 plays a role in ERK activation during inflammatory and stress responses and has been implicated in the onset of arthritis [77]. KSR1-facilitated ERK activation was also found to be essential for long-term memory formation and synaptic plasticity [78].

KSR-1 association with MEK kinases is constitutive, while binding to Raf and recruitment of ERK to the complex are Ras activation-dependent. Another protein constitutively bound to KSR1 and required for maximal stimulation the ERK cascade is casein kinase 2 [79]. KSR1 is able to interact with Gβγ *via *the γ subunit and is recruited to the plasma membrane upon LPA stimulation ([80]; Fig. 5B).

JNK-interacting leucine zipper protein (JLP) is a scaffolding protein (recently reviewed in [72]) involved in linking JNK with its substrate transcription factors (Myc and Max) and its upstream kinases (MKK4 and MEKK3) [81]. JLP interacts with kinesin light chain 1, which is required for its interaction with JNK and affects localization of the complex [82]. JLP has recently been found to interact specifically with Gα_12 _and Gα_13_, but not Gα_s_, Gα_i _or Gα_q _[83]. Although JLP binds both activated and inactive Gα_13_, the interaction is more pronounced with the activated form. Accordingly, JLP-Gα_13 _interaction is enhanced by lysophosphatidic acid (LPA) stimulation, which is known to activate Gα_13_. LPA-induced JNK activation in living cells is potentiated by overexpression of JLP. Thus, JLP appears to function in linking GPCR signaling to JNK activation by assembling a protein complex that involves Gα_12 _or Gα_13 _and the components of JNK signaling module ([83]; Fig. 5C). We have recently found that JLP can also assemble a complex containing Gα_13 _and apoptosis signal-regulating kinase 1 (ASK1), which is known to stimulate JNK and/or p38 in response to stress stimuli [84]. Although the functionality of this complex in terms of activation of downstream MAP kinases remains to be tested, this suggests that JLP may link Gα_12/13 _signaling with JNK through more than one kinase cascade. A physiological role for the Gα_13_-JLP-JNK signaling complex in retinoic acid-induced differentiation has recently been suggested [85].

Two JLP-related alternative splicing products are known (JIP4 and SPAG9), which differ from JLP in their N-termini and appear to have different selectivity towards JNK *vs*. p38 [86,87]. Since Gα_12/13_-binding region in JLP is at the C-terminus and the C-terminal parts are conserved in all three isoforms, JIP4 and SPAG9 would also likely be able to interact with Gα_12/13_. Experimental confirmation of this interaction and its functional implications have not yet been reported.

## Conclusion and perspectives

A number of scaffolding proteins for heterotrimeric G proteins have been identified in the recent years, and undoubtedly many more are still to be found. The knowledge of the composition of possible complexes formed by these scaffolds is essential for identification of the precise signaling pathways they may be involved in. This has been already achieved to some extent for a number of signaling complexes that involve heterotrimeric G proteins. Another layer of complexity is added by the events that determine when and at what levels these scaffolds are expressed in the cell and what are the fine tuning mechanisms that regulate their interactions with particular partners. In this respect, recent studies have highlighted some possible fine tuning mechanisms. First, the expression of scaffolding proteins may be regulated by relevant physiological stimuli, as evidenced by the work of Kashef *et al*. [85], who found that retinoic acid may induce expression of JLP, a scaffolding protein that assembles a complex transducing the signal from this ligand. Another example of regulation of a G protein-binding scaffold by a physiological stimulus, in this case growth factor stimulation [76], is Ras-dependent degradation of a protein IMP that controls KSR1 localization [88]. Another level of fine tuning may be achieved by posttranslational modifications of the proteins involved, and some cases of such regulation are beginning to emerge. It was suggested that binding of Gβγ may Ste5 may induce conformational changes in the latter, which make activation of MAP kinases bound to Ste5 more efficient [89]. Regulation of several G protein-binding scaffolding proteins by phosphorylation has been reported, including Ste5 and KSR1 [76,89]. Sumoylation of the proteins of RGS-Rz subfamily was found to switch their function from stimulation of GTPase activity of Gα subunits to that of a scaffolding protein [90]. Clearly, detailed knowledge of not only composition of the complexes assembled by G protein-interacting scaffolding proteins, but although intricate regulation of the properties of all components of these complexes will be necessary for full understanding of their biological functions.

## Competing interests

The author(s) declare that they have no competing interests.
